# Small Copy Number Neutral Intrachromosomal Translocation of *PAX6* and Aniridia

**DOI:** 10.1001/jamaophthalmol.2026.1389

**Published:** 2026-05-14

**Authors:** Linda M. Reis, Jared Tomei, Ryan Gallagher, Andrea Matter, Joseph Carroll, Ulrich Broeckel, Elena V. Semina

**Affiliations:** 1 Department of Ophthalmology and Visual Sciences, Medical College of Wisconsin, Milwaukee; 2 Department of Pediatrics, Medical College of Wisconsin and Children’s Wisconsin, Milwaukee; 3 Department of Cell Biology, Neurobiology and Anatomy, Medical College of Wisconsin, Milwaukee

## Abstract

**Importance:**

Approximately 5% to 10% of individuals with classic aniridia do not receive a molecular diagnosis after clinical testing for variants in *PAX6* and its downstream regulatory region.

**Objective:**

To apply optical genome mapping (OGM) and long-read whole-genome sequencing (lrWGS) to diagnose an individual with unexplained classic aniridia.

**Design, Setting, and Participants:**

High-quality DNA was extracted from the blood of a 16-year-old male patient with classic aniridia and prior negative clinical test results that included sequencing and copy number analysis of *PAX6* exons and downstream regulatory region as well as genomic analysis via short-read whole-genome sequencing (srWGS) and analyzed using OGM and lrWGS. All analyses were performed in a research laboratory in Wisconsin from January 2019 to September 2025.

**Interventions:**

OGM and lrWGS.

**Main Outcomes and Measures:**

Identification of a structural variant disrupting *PAX6* expression in an individual with classic aniridia, following negative prior testing including srWGS.

**Results:**

OGM identified a 55-kb deletion on 11p13 encompassing all *PAX6* exons and exon 12 of *ELP4*, with insertion of this segment into 11q21. lrWGS delineated the exact breakpoints, confirming that the downstream regulatory region, required for normal *PAX6* expression, remained at the 11p13 locus. Consequently, the translocated copy of *PAX6* at 11q21 is expected to lack expression due to the loss of its essential regulatory elements.

**Conclusions and Relevance:**

These findings in an individual with classic aniridia harboring an intrachromosomal rearrangement at the *PAX6* locus identified by OGM and lrWGS may represent the smallest reported structural variant to separate the *PAX6* coding sequence from its downstream regulatory region. This structural variant may have fallen below the detection threshold of srWGS due to its balanced nature and small size, suggesting OGM and lrWGS would be needed for definitive identification.

## Key Points

**Question:**

What is the genetic basis of classic aniridia in an individual with negative *PAX6* clinical test results along with short-read whole-genome sequencing (srWGS)?

**Findings:**

This diagnostic study combining optical genome mapping (OGM) and long-read whole-genome sequencing (lrWGS) revealed a novel small balanced intrachromosomal translocation that included all *PAX6* exons but separated the gene from its downstream regulatory region critical for expression.

**Meaning:**

These results suggest OGM and lrWGS can overcome the diagnostic limitations of srWGS in detecting small balanced translocations, potentially providing a strategy for solving unexplained aniridia, with a specific focus on the *PAX6/ELP4* region.

## Teaser

This diagnostic study aims to determine whether optical genome mapping and long-read whole-genome sequencing can identify a pathogenic structural variant disrupting *PAX6* in an individual with classic aniridia after negative standard clinical and short-read genomic testing.

## Introduction

Classic aniridia is a panocular disease characterized by absent or hypoplastic iris, foveal hypoplasia, and high rates of cataracts, glaucoma, and/or corneal keratopathy. Approximately 90% to 95% of aniridia is caused by *PAX6* haploinsufficiency, including intragenic variants, gene deletions, or deletions affecting a downstream regulatory region (DRR) within the neighboring *ELP4* gene.^1,2^ The monogenic nature of typical aniridia provides an opportunity to identify novel mechanisms through further investigation of *PAX6*-negative cases. This study used 2 new platforms, optical genome mapping (OGM)^3^ and long-read whole-genome sequencing (lrWGS),^4^ to investigate unexplained aniridia in an affected individual and identified a complex balanced intrachromosomal rearrangement.

## Methods

The Medical College of Wisconsin institutional review board approved this study with written informed consent. Short-read whole-genome sequencing (srWGS) was performed and processed using standard research protocols and bioinformatics pipelines (eMethods in Supplement 1). OGM (Bionano) and lrWGS (Oxford Nanopore Technologies) were performed as described^3^ and to an average coverage of 249.92× and an average fragment length of 334.14 kb for OGM and to a target coverage of 52× (average single read length of 26 kb) for lrWGS (eMethods in Supplement 1). Integrative Genomics Viewer was used to verify breakpoints and haplotype information.

## Results

**Figure 1.**
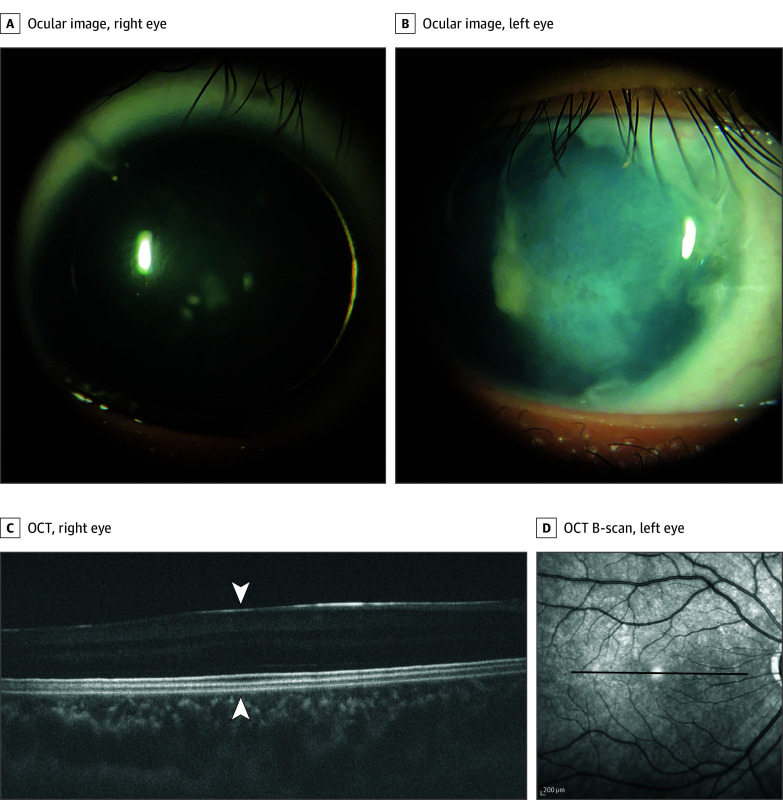
Ocular Images and Optical Coherence Tomography (OCT) Showing Typical Aniridia Ocular features include absent iris (A) and corneal opacification (B). OCT imaging revealed grade 2 foveal hypoplasia, with subtle cone outer segment elongation and extrusion of the inner retinal layers present at the location of the incipient fovea (C, arrowheads). The position of the OCT B-scan on the fundus is shown by a solid line (D).

**Figure 2.**
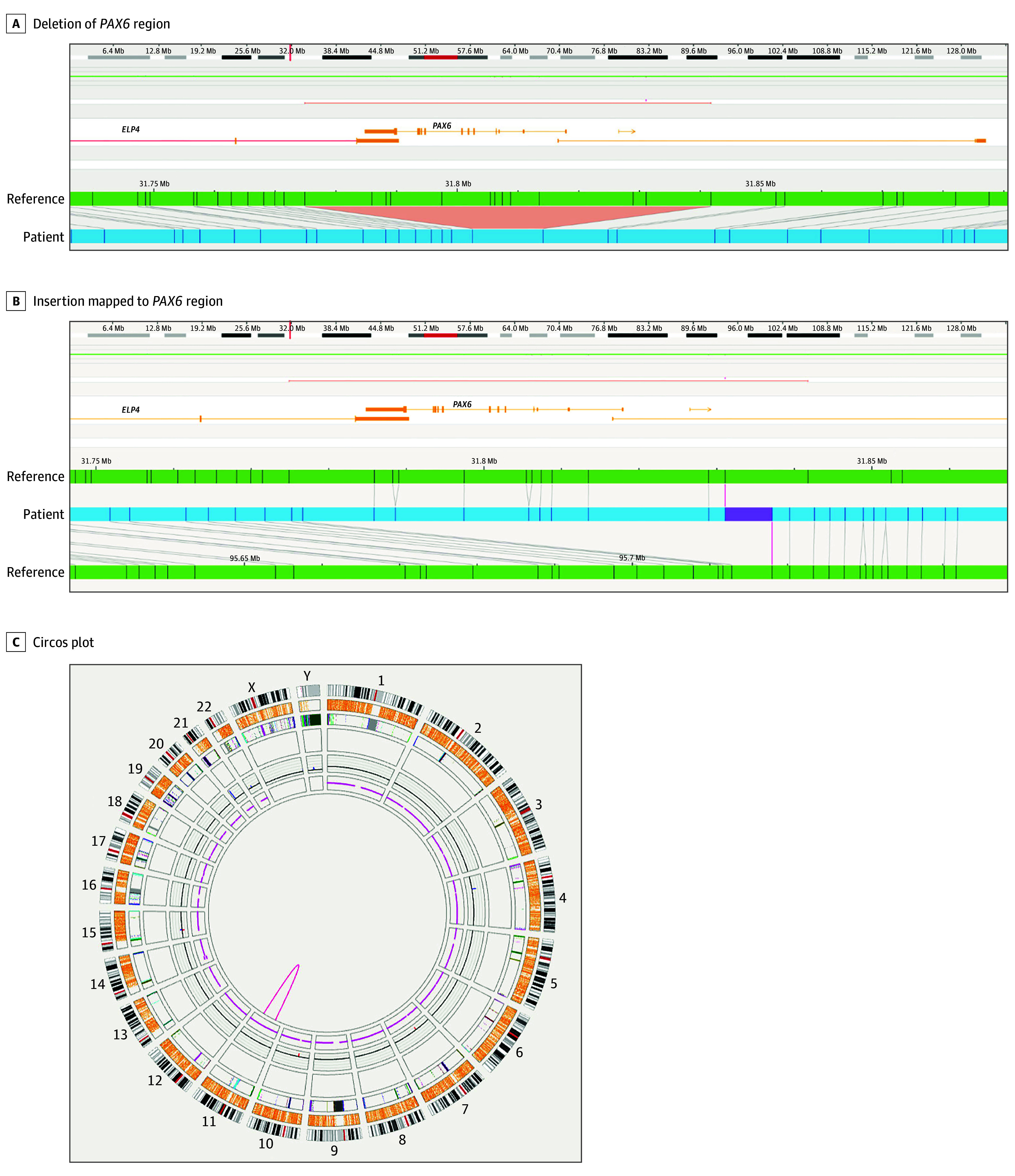
Schematic Representations and Circos Plot Showing Intrachromosomal Deletion and Insertion Intrachromosomal insertion as visualized by Bionano optical genome mapping (OGM). Images show deletion of the *PAX6* region via a missing segment on the patient track (A) and intrachromosomal insertion of a segment on the other arm with inserted labels mapping to the *PAX6* region (B). Circos plot (C) of the entire genome shows a U-shaped line on chr11 indicating the intrachromosomal insertion.

A 16-year-old adopted male patient with typical aniridia (Figure 1) including absent iris, posterior polar cataract, keratopathy, foveal hypoplasia, nystagmus, and glaucoma, and negative comprehensive clinical test results for *PAX6* enrolled in the research study to identify a genetic cause; srWGS did not identify any causative variants. Copy number analysis of srWGS data identified a low-confidence deletion call within the *PAX6/ELP4* region; however, the presence of 52 of 65 heterozygous variants within the candidate deletion region did not support the deletion call in this region. OGM detected 2 events relevant to the *PAX6* region: a heterozygous 55-kb deletion on 11p13 encompassing all of *PAX6* and exon 12 of *ELP4* (estimated coordinates of 11:31774854-31841770) and an intrachromosomal insertion of the same 55 kb into 11q21 estimated at chr11:95 712 800. In combination, the findings identify a copy number neutral intrachromosomal translocation where *PAX6* is removed from 11p13 and inserted into 11q21 (Figure 2). lrWGS determined the precise breakpoints for the translocated segment as chr11:31 779 025 and chr11:31 834 240 and for the insertion location as chr11:95 715 120 (eFigure in Supplement 1). Based on these breakpoints, the DRR region (chr11:31 626 701-31 644 792) required for *PAX6* expression remained in its usual genomic context. Since the DRR is now more than 63 Mb from the translocated *PAX6* gene, the translocated copy of the gene is expected to not be expressed,^5^ resulting in *PAX6* haploinsufficiency, the primary mechanism underlying aniridia.^1^

## Discussion

While there is an increasing recognition that OGM and lrWGS can detect previously difficult-to-identify structural variants due to their greater resolution, to the best of our knowledge a clinical phenotype caused by a balanced rearrangement involving an intrachromosomal translocation of all exons of a single gene of this size has not yet been reported. Thus, this report presents a case with an intrachromosomal rearrangement specifically affecting the *PAX6* gene and may represent the smallest rearrangement known to separate *PAX6* from the DRR. This structural variant may have fallen below the detection threshold of srWGS due to its balanced nature and small size, suggesting OGM and lrWGS would be needed for definitive identification.

### Limitations

These findings are based on 1 individual. Additional cases would be needed to validate the conclusions.

## Conclusions

These findings further highlight the technical abilities of OGM, which identified and characterized the rearrangement. In combination with lrWGS, which ascertained the breakpoints, we were able to define this complex chromosomal rearrangement. These findings support use of OGM and lrWGS for unexplained typical aniridia, with a specific focus on the *PAX6*/*ELP4* region.

## Notes

Supplement 1. eMethods. Additional Material and Methods eFigure. Intrachromosomal insertion breakpoints as visualized by long read WGS

Supplement 2. Data sharing statement
