# Supplementary material for: Small Copy Number Neutral Intrachromosomal Translocation of PAX6 and Aniridia
Source: JAMA Ophthalmol. 2026 May 14;144(6):541–4. doi: 10.1001/jamaophthalmol.2026.1389 (PMC13177189; doi:10.1001/jamaophthalmol.2026.1389)
Supplement: Supplement 1. — eMethods. Additional Material and Methods eFigure. Intrachromosomal insertion breakpoints as visualized by long read WGS [file jamaophthalmol-e261389-s001.pdf]

Supplemental Online Content

Reis LM, Tomei J, Gallagher R, et al. Small copy number neutral intrachromosomal translocation of *PAX6* and aniridia. *JAMA Ophthalmol*. Published online May 14, 2026. doi:10.1001/jamaophthalmol.2026.1389

eMethods. Additional Material and Methods
eFigure. Intrachromosomal insertion breakpoints as visualized by long read WGS

This supplemental material has been provided by the authors to give readers additional information about their work.

© 2026 Reis LM et al. JAMA Ophthalmology.

eMethods

The patient was adopted from China with no family history information available. Clinical testing was completed in 2014 including Sanger sequencing of all exons of *PAX6* for intragenic variants and targeted exon level array CGH analysis of *PAX6, DCDC1, ELP4,* and *WT1.* Short read whole genome sequencing (srWGS) was performed by Psomagen and processed using standard bioformatics pipelines aligned to build hg19. Fastq files were re-processed by Mayo Genome Core using their in-house Mayo GenomeGPS pipeline as previously described[1] and aligned to build hg38; Manta[2], GRIDSS[3], and CNVPytor[4] were applied for copy number calling. VCF and BAM files were analyzed using Golden Helix VarSeq and Genome Browser software (Bozeman, MT) including copy number caller as previously described[5].

For Bionano Optical Genome Mapping (OGM), samples were processed as recently described[6]. In brief, ultra high molecular weight (HMW) genomic DNA was extracted using Bionano's SP-G2 Blood & Cell Culture DNA Isolation Kit, followed by direct labeling of the gDNA using the Direct Label and Stain-G2 kit. The sample was loaded onto a Bionano chip and ran on the Bionano Saphyr for 18 hours. Subsequent de novo genome assembly was conducted using the Bionano Access 1.8 software and Bionano Solve 3.8 pipeline. Structural variants were identified in comparison to the reference sequence (hg38) using the standard Bionano analytic pipeline and inbuilt recommended filter system in which common or technical variants are filtered out, leaving only high confidence and rare (<1% in the Bionano Control Database for any sample and samples labeled with the same Enzyme) Structural Variants for interpretation. Variants were filtered to be within 12kb of a gene on the reference. OGM was performed to an average coverage of 249.92x and an average fragment length of 334.14 kb.

Long Read WGS was performed using Oxford Nanopore Technologies (ONT) platform. HMW genomic DNA was extracted in triplicate from frozen whole blood using the Nanobind CBB Kit according to the manufacturer's recommendations (PacBio). 8 µg of HMW DNA was sheared using a Megaruptor 3 Shearing Kit on a

© 2026 Reis LM et al. JAMA Ophthalmology.

Megaruptor 3 DNA Shearing System using the standard protocol with a speed of 18 (Hologic Diagenode). Sheared HMW DNA was concentrated to 60 µl and was subjected to short read elimination using a SRE kit following manufacturer's instructions (PacBio). Library Preparation was performed in triplicate using 3 µg DNA input with the Ligation Sequencing Kit (ONT) and the NEBNext Companion Module for Oxford Nanopore Technologies Ligation Sequencing (New England Biolabs). Sequencing was performed on a PromethION 24 instrument using a R10.4.1 flow cell (ONT) with the base calling model set to Super-Accurate and the run limit changed to flow cell end of life. During the run, the flow cell was washed using a Flow Cell Wash Kit (ONT) and reloaded with remaining library twice when <25% of the active pores were remaining. Base calling occurred using MinKnow (v24.06.15) live base-caller for ONT Dorado (v.7.4.14). Sequence alignment was performed using MiniMap2 (v2.24) against human GRCh38. Analysis was performed using IGV (v.2.19.3) to display variants called at the base-pair level with Pepper-Margin-DeepVariant (v.0.8) and at the structural level with Sniffles2 (v.2.4) and ont-spectre (v.0.2.2). Proper phasing for regions of interest was validated via phaseset data. IGV was used to verify breakpoints and haplotype information. We performed long read sequencing to a target coverage of 52x with an average single read length of 26 kb.

Relevant aspects of STARD reporting guidelines were followed for this manuscript.

References:

1. Vasmatzis G, Liu MC, Reganti S, et al. Integration of Comprehensive Genomic Analysis and Functional Screening of Affected Molecular Pathways to Inform Cancer Therapy. *Mayo Clin Proc*. Feb 2020;95(2):306–318. doi:10.1016/j.mayocp.2019.07.019
2. Chen X, Schulz-Trieglaff O, Shaw R, et al. Manta: rapid detection of structural variants and indels for germline and cancer sequencing applications. *Bioinformatics*. Apr 15 2016;32(8):1220–2. doi:10.1093/bioinformatics/btv710
3. Cameron DL, Schroder J, Penington JS, et al. GRIDSS: sensitive and specific genomic rearrangement detection using positional de Bruijn graph assembly. *Genome Res*. Dec 2017;27(12):2050–2060. doi:10.1101/gr.222109.117
4. Suvakov M, Panda A, Diesh C, Holmes I, Abyzov A. CNVpytor: a tool for copy number variation detection and analysis from read depth and allele imbalance in whole-genome sequencing. *Gigascience*. Nov 18 2021;10(11)doi:10.1093/gigascience/giab074
5. Reis LM, Atilla H, Kannu P, et al. Distinct Roles of Histone Lysine Demethylases and Methyltransferases in Developmental Eye Disease. *Genes (Basel)*. Jan 14 2023;14(1):216. doi:10.3390/genes14010216
6. Broeckel U, Iqbal MA, Levy B, et al. Detection of Constitutional Structural Variants by Optical Genome Mapping: A Multisite Study of Postnatal Samples. *J Mol Diagn*. Mar 2024;26(3):213–226. doi:10.1016/j.jmoldx.2023.12.003

© 2026 Reis LM et al. JAMA Ophthalmology.

eFigure 1: Intrachromosomal insertion breakpoints as visualized by lrWGS. Images from sorted alignment showing leftmost breakpoint on 11p13 (A), rightmost breakpoint on 11p13(B), and the insertion breakpoint on 11q21 (C) along with the full deletion region (D) and insertion location (E) sorted by haplotype grouping.

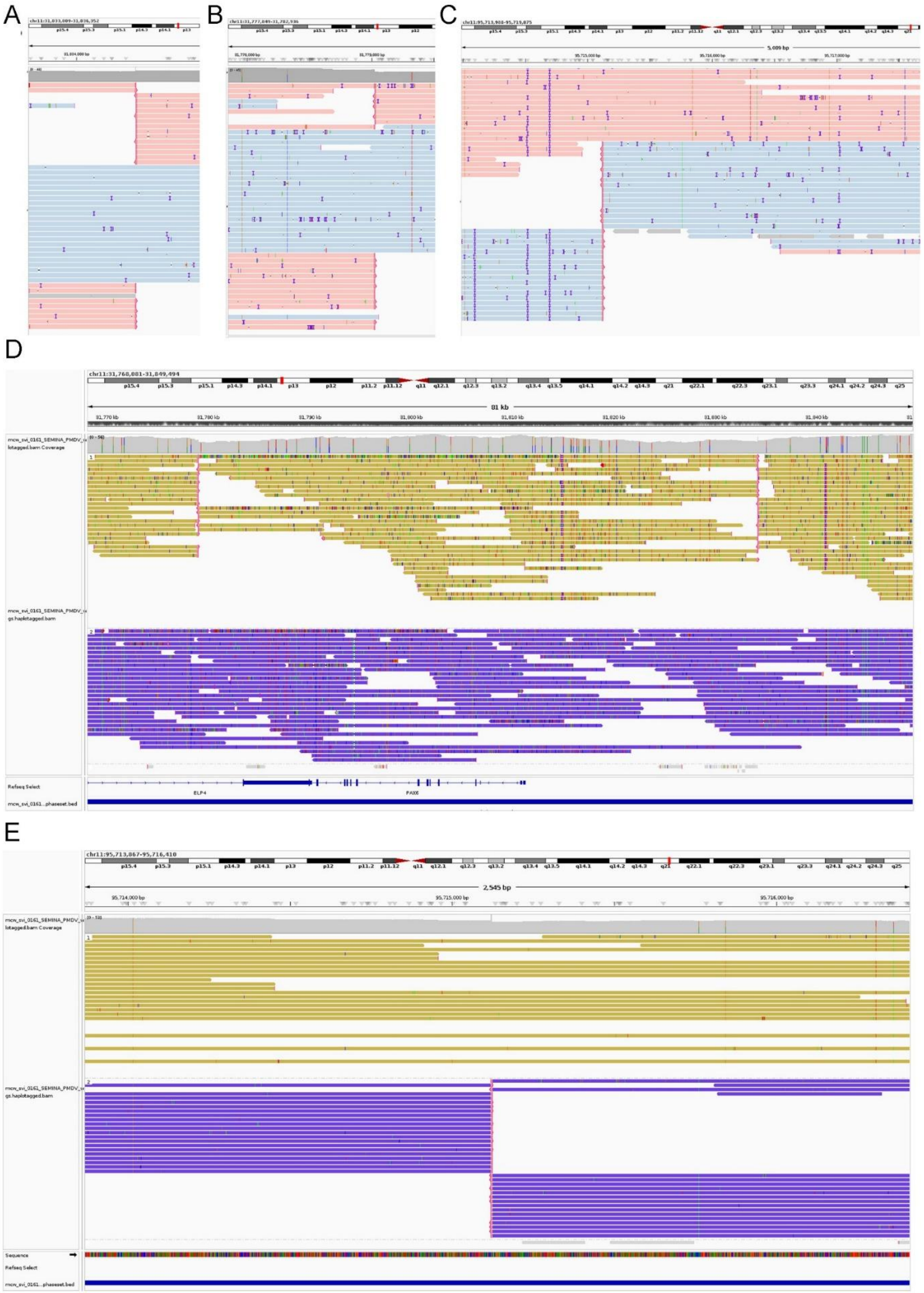

© 2026 Reis LM et al. JAMA Ophthalmology.
