# Supplementary material for: Small Copy Number Neutral Intrachromosomal Translocation of PAX6 and Aniridia
Source: JAMA Ophthalmol. 2026 May 14;144(6):541–4. doi: 10.1001/jamaophthalmol.2026.1389 (PMC13177189; doi:10.1001/jamaophthalmol.2026.1389)
Supplement: Supplement 2. — Data sharing statement [file jamaophthalmol-e261389-s002.pdf]

Data Sharing Statement

Reis. Small Copy Number Neutral Intrachromosomal Translocation of PAX6 and Aniridia.
JAMA Ophthalmol. Published May 14, 2026. doi:10.1001/jamaophthalmol.2026.1389

Data
Data available: Yes
Data types: Deidentified participant data
How to access data: https://www.ncbi.nlm.nih.gov/clinvar/
When available: With publication

Supporting Documents
Document types: None

Additional Information
Who can access the data: Who can access the data: anyone requesting the data
Types of analyses: Types of analyses: Clinical variant classification
Mechanisms of data availability: Mechanisms of data availability: The variant data on ClinVar will be available publicly
Any additional restrictions: None
